# Everybody's Page

**Published:** 1888-02-18

**Authors:** 


### Everybody's Page.

The statistics of the export of Japanese tea show that the quantity annually sent abroad is increasing, but the prices are falling lower. America is Japan's chief customer at present. In 1859, the first year Japan was opened to foreign trade, the export was 20,000 lbs., while last year it reached 22,500,000 lbs.

---

An alkaline extract of coal has been obtained by P. E. Reisch, by digesting coal dust with caustic soda, boiling the liquid, and afterwards neutralizing it with hydrochloric acid. In this manner he obtains a new tanning material which he calls pyrofuscine; and though the process of treating leather with it is more complicated than the usual tanning process, he believes it to be half as cheap as the bark process, and cheaper than the alum process.

---

As illustrative of the means by which some sorts of cheap German goods are produced, a factory inspector states that at Düsseldorf children of little over four years of age were found helping in the weaving factories. The law prohibits children under twelve years of age being employed, but apparently it is being disobeyed with impunity.

---

A very ingenious invention is the "Immisch" clinical thermometer. It is in appearance like a small watch, and can be attached to an ordinary watch chain, which makes it specially suitable for the use of travellers; and in sensitiveness and accuracy it has been proved to be quite equal to the thermometer of conventional shape. The "Immisch" thermometer received the first-class award for accuracy and portability at the International Medical Congress in 1881.

---

It was once our fortune to sit at a card-table where a young lady was receiving her first lesson in the noble science of whist from an elderly gentleman of somewhat irascible temper. When the game was finished—she had hoarded her trumps, forgotten her partner's lead, thrown away the opportunity for a "cross-ruff," and committed other deadly sins—she asked, in tones of modest self-satisfaction, "Do you think I shall ever make a good whist-player?" Her instructor glared at her for a moment, and answered curtly, "No; wrong sex." What would this old gentleman, who held whist to be a thing beyond the grasp of the feminine intellect, have thought if he had known that among the many occupations that women have lately taken up is that of teaching whist! In New York several ladies earn a living in this way. It is to be hoped that they "turn out" creditable pupils; though for our own part we are strongly of opinion that the whist-player, like the poet, is born, not made.

---

The latest limited liability company is one which insures hats and umbrellas, so that if the one is lost and the other "taken by mistake," the owner can always rely upon a new article in its place. The prospectus of the company states that "among the smaller ills for which civilisation has failed to supply a remedy is the vexation caused by the loss of a hat or umbrella." By payment of a moderate premium it will be possible to secure the possession of a new hat and umbrella at the cost of the company and without expense or trouble to the assured. The insurer will receive his policy in the form of a card, which can be easily carried in the pocket. The company will appoint hatters and umbrella-makers in all parts of London and the chief towns of the United Kingdom as its agents. As soon as an insurer sees his hat on the waves or misses his umbrella, he simply goes to the company's agent, and is supplied with a brand new one. The worst part of it is, with regard to the loss of the hat, that the insurer must go hatless before a Commissioner of Oaths and make a statutory declaration about his loss.

The British agriculturist may be poor in all else, but he is rich in advisers. The counsel they give is often so contradictory that in sheer bewilderment he tends to prefer walking in his old paths. Among other suggestions which have been made to him is that he should cultivate flax. There is a continual demand for flax in our linen factories; there is plenty of land in the country suited for growing flax; yet almost all the fibre employed in this country is imported from the Continent. Is not this a case of paying other people to do what we could do as well ourselves?

---

An interesting *café* and *brasserie* has been recently opened in Montmartre, Paris. It is built in the form of a gigantic tub, and the signboard is dedicated "To Diogenes." But Diogenes did not receive visitors in his tub; in fact he took up his abode there because he was disgusted with mankind, and wanted to avoid them all, except perhaps his brother stoics. Surely the proprietors of the Café Diogène do not wish to repel customers, and it is not the stoic who as a rule frequents the public-house. The owners of the new *brasserie* have read their classical dictionary too carelessly.

---

Iodoform is recommended by Messrs. Casson, M.R.C.S., and Bronnen, F.C.S., to be combined with candles to form a sort of automatic disinfectant. The power of iodine in this respect is well known. When iodoform is heated it decomposes, iodine being one of the products; but as this does not take place regularly the iodoform is combined in the candles with salicylic acid, so that while the candles are burning both iodine and phenol are given off. These candles have been found useful for the sick rooms of those suffering from asthma, spasmodic cough, and hay fever. The air of "stuffy" rooms is also rapidly purified by means of them.

---

The digestibility of cod liver oil, like that of cream and butter, depends upon what it is made of. In a journey to Norway a great deal can be learned about cod liver oil, and the reason why some people hold it in such repugnance becomes very apparent. It is only necessary to touch on the manufacture of the pure oil, without complicating the matter with the question of adulteration with olive oil and other things, which, unfortunately, exists largely. The old method of manufacturing cod liver oil, by leaving the livers in barrels to ferment until the oil vessels in them shall burst and the oil float to the top of the barrel, is still largely carried on; and this "raw" or natural oil is what is generally used in Germany and parts of France to this day. It varies in colour from light straw to a deep brown, contains a large amount of oleic acid, and besides being unpalatable, is exceedingly difficult of digestion, owing to the amount of stearine it contains. For some years now the "steam" or cooked oil has been steadily taking the place of the natural or raw oils, and to prepare this the livers must be as fresh as it is possible to get them, and the oil is extracted in steam "jacketed" pans. This process produces a light, golden-coloured oil, perfectly sweet to the taste if only fresh livers are used. To get these as quickly as the fish are caught is the first essential. For this purpose Messrs. Jensen, the largest manufacturers of cod liver oil, whose "fabriks" for oil refining and fish guano at Brettesnoes employ 700 hands, have a large 1,000-ton steamer fitted up as a factory. This vessel follows the fleet of fishing boats in order to get the livers as soon as the fish are caught. Steam oil made from fresh livers requires only freezing to get out all the stearine or indigestible matter, and then, after being carefully filtered, is ready for bottling. If properly manufactured in this way, steam oil is as digestible as fresh butter, and quite as palatable; but unless the oil is extracted from livers perfectly fresh, no refining can make it taste sweet. The livers are too often packed into barrels and kept for days before the buyers from the factories at Bergen get them; in which case the livers are tainted and the oil suffers.

